# Precipitates and Particles Coarsening of 9Cr–1.7W–0.4Mo–Co Ferritic Heat-Resistant Steel after Isothermal Aging

**DOI:** 10.1038/s41598-017-06191-2

**Published:** 2017-07-19

**Authors:** Qiuzhi Gao, Yanan Zhang, Hailian Zhang, Huijun Li, Fu Qu, Jian Han, Cheng Lu, Bintao Wu, Yao Lu, Yan Ma

**Affiliations:** 1 School of Materials Science and Engineering, Northeastern University, Shenyang, 110819 China; 2 Research and Development Department, CITIC Bohai Aluminium Industries Holding Company, LTD., Qinhuangdao, 066000 China; 3 School of Economics and Management, Yanshan University, Qinhuangdao, 066004 China; 4 School of Mechanical, Materials and Mechatronic Engineering, University of Wollongong, Wollongong, NSW 2522 Australia

## Abstract

The precipitates obtained by EPE technology from the 9Cr-1.7W-0.4Mo-Co ferritic heat-resistant steel subject to isothermal aging were investigated using SEM, TEM and XRD. The particle size distribution and the coarsening kinetics of M_23_C_6_ with duration of isothermal aging were also analyzed with or without consideration of Laves phase. The results show that the isolated dislocations were detected in delta ferrite interior, and the precipitates on delta ferrite and martensite boundaries are obviously larger than other locations. Fe_2_W-Laves phase can only be found as duration of aging time to 2000 h, and is preferential to form adjacent to M_23_C_6_ particles. The small M_23_C_6_ particles firstly coarsen, but the large M_23_C_6_ are relatively stable during short aging. The total coarsening rate of M_23_C_6_ precipitates is 9.75 × 10^−28^m^3^s^−1^, and the coarsening of M_23_C_6_ depends on the formation of Laves phase.

## Introduction

9~12wt.% Cr ferritic creep steels, as the key materials increasing the thermal efficiency of steam power plants, are widely used in power industries due to its excellent high temperature stability, high creep strength and stress corrosion cracking^1–4^. The excellent properties have been achieved through apparently minor compositional changes to well established steels like P91, P92 and P122^5, 6^. However, the main problem of 9~12 wt.% Cr ferritic steels that has been addressed is that their microstructural instability with a degradation in oxidation resistance during long term service at temperature up to 923 K^7^. New alloys, such as NF12 and SAVE12, based on optimization of C, Mo and W, partial substitution of Mo by W, and addition of Co, Ta, Nd, has been developed to allow the application above 923 K^6, 8^.

Microstructure of 9~12 wt.% Cr ferritic creep steels, as generally composed by tempered martensite matrix and 6~8% delta ferrite, is stabilized by solution strengthening and precipitation strengthening. During long term aging at high temperature, the microstructural evolutions can be described as follows: recovery of martensite matrix (growth of martensitic lath), coarsening of precipitates (M_23_C_6_), precipitation of new brittle intermetallic phases (Laves phases, Z-phase) on grain boundaries and subgrain (martensite lath) boundaries^9–12^. The coarsening of precipitates and the precipitation of new brittle intermetallic phases are deemed to result in the microstructural instability, deteriorate the creep strength and the fracture properties. M_23_C_6_ second phase is considered to mainly precipitates on prior austenite grain boundaries and martensitic subgrain boundaries, and act as an obstacle of boundaries migration^13^. However, the coarsening of M_23_C_6_ particles tends to be easy during aging due to the large solubility of Fe and Cr as the major composed elements of M_23_C_6_. M_23_C_6_ particles are general considered to play a major role on the stabilization of the tempered martensite lath structure, comparing to MX and Laves phase, and thus, prevention of coarsening of M_23_C_6_ particles is important for creep strength improvement^14, 15^.

Laves phase, composed by [(Fe, Cr)_2_ (W, Mo)], only appears during aging/creep^16, 17^. The precipitation of Laves phase during long term aging leads to a depletion of W and Mo as the solid solution strengthening elements in the matrix, and, thereby, to a reduction of the solid solution strengthening in 9~12 wt.% Cr ferritic creep steels. Whereas, Laves phase with small particle size also can increase the creep strength by precipitation hardening at early stage of aging/creep^18–20^. In addition, compared to M_23_C_6_ and Laves phase, MX carbonitrides (i.e., M = metals, X = C/N) exhibit the smallest size, and mainly distribute at subgrain boundaries and within martensite laths^21, 22^. Chen *et al*.^23^ showed that martensite laths with the finely dispersed nanosized MX in 9 wt.% Cr ferritic steel would contribute to the improvement of high-temperature mechanical performance. Panait *et al*.^22^ displayed that MX carbonitrides are very stable during thermal and creep exposure of P91 steel at 873 K up to 10^5^ h.

According to Zener pinning equation, the pinning effort of the particles is related to particle size and distance between each other^24, 25^. Based on this, the pinning forces of MX carbonitrides should be higher than that of M_23_C_6_ carbides. Furthermore, Some references^26, 27^ also proved that MX carbonitrides in 9 wt.% Cr ferritic steel were more advantageous than M_23_C_6_ carbides because MX precipitated finely and densely, and did not coarsen easily at high temperatures. Factually, MX carbonitrides effectively strengthen boundaries because they can act as obstacles to migrating boundaries^28^, and are very stable during short aging/creeping. Thus, in the case of without considering the evolution of MX carbonitrides, the coarsening of M_23_C_6_ particles and the formation of Laves phase, both as the key factors that will affect the microstructural stability of 9~12 wt.% Cr ferritic creep steels during aging/creep, especially 9 wt.% Cr creep strengthening steel with addition of Co, should be given more enough data to find ways for improving the aging/creep strength of the steels.

The content of delta ferrite in 9~12 wt.% Cr ferritic creep steels can be influenced by the alloy elements as W, Mo, Co, *etc*., especially as C^29^. The diagram of transformed phase (delta ferrite) as a function of net chromium equivalent (NCE) has been characterized in some references^29, 30^. For example, there is no delta ferrite in T91 ferritic steel, but it is up to 6~8% in modified ferritic steel with Co addition and elements adjustment^31, 32^. With the situation of high content, delta ferrite plays important roles on solid solution strengthening of alloying elements and precipitation behavior of secondary phases, such as M_23_C_6_ and Laves phase. The dissolved alloying elements (such as Cr, C, W, Mo, and so on)contents in delta ferrite matrix are theoretically higher than that in martensite for the tempered samples, which will promote the formation of the precipitates^33–35^. Whereas, the solidifications of other alloying elements, such as Co, Mn and Ni, are crucial in hindering the formation of delta ferrite^33, 36^. The works in our previous researches also proved that the content Redjaimia *et al*.^37^ showed that the precipitation of M_23_C_6_ phase priors to the other precipitates on delta ferrite boundaries, which is always attributed to the high mobility of carbon towards the boundaries enriched in M_23_C_6_ forming alloying elements. In addition, accompanying with precipitation of secondary phases, the structure of delta ferrite has been changed, and some dislocations should be found in the interior of delta ferrite, which is rarely reported and explained.

The microstructural evolution and the distribution of alloying elements of the new modified 9Cr-1.7W-0.4Mo-Co ferritic heat-resistant steel during isothermal aging were described in our previous article^35^. This article presents a detailed description of the precipitation behavior in the same aged steel. The powder of the precipitates is obtained by electrolytic phase extraction (EPE) technology, and analyzed using scanning electron microscope (SEM), transmission electron microscopy (TEM) and X-ray diffraction (XRD). M_23_C_6_ and Laves phase were identified by the selected area diffraction (SAD). The particle size distribution and the coarsening kinetics of M_23_C_6_ precipitates were also analyzed.

## Results

### Microstructural evolutions on delta ferrite boundaries

**Figure 1:**
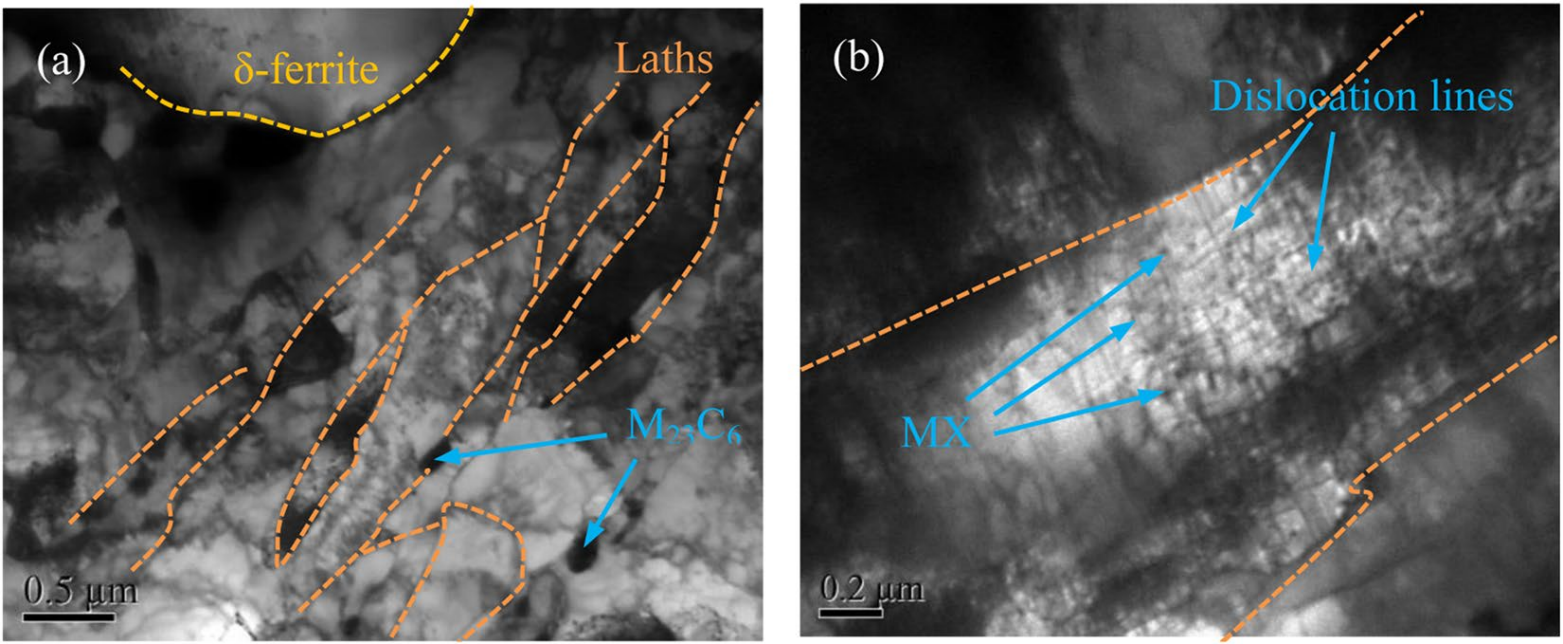
TEM micrographs of 9Cr-1.7W-0.4Mo-Co ferritic steel after isothermal aging for 500h, total microstructure (**a**) and details in martensite lath (**b**).

TEM micrographs of 9Cr-1.7W-0.4Mo-Co ferritic steel aged for 500 h are displayed in Fig. 1. Martensite consists of laths and delta ferrite can be distinguished, and some M_23_C_6_ precipitates with large particles distributed at martensite lath boundaries and the interface of delta ferrite and martensite. In addition, nano-sized MX precipitates were homogeneously distributed in martensite lath, which would impede the migration of high-density dislocations and insist stable microstructure during isothermal aging (seen in Fig. 1b).

As we discussed in our previous works^35, 38^, there were many precipitates particles located at the interface of delta ferrite and martensite in ferritic heat-resistant steels. The delta ferrite, enriched in BCC stabilising alloying elements, notably Cr, Mo and W, is a favourable site for some precipitates, such as M_23_C_6_, Laves phase, comparing to austenite and martensite. Therefore, it means that the alloying elements, which are contributed to the formation of precipitates located at the boundaries of delta ferrite grain, are provided by delta ferrite phase. During aging, the migrations of alloying elements from the interior to the grain boundaries of delta ferrite phase lead to the formation of precipitates with a large number, and new dislocations located in the interior of delta ferrite phase might be emerged.

**Figure 2:**
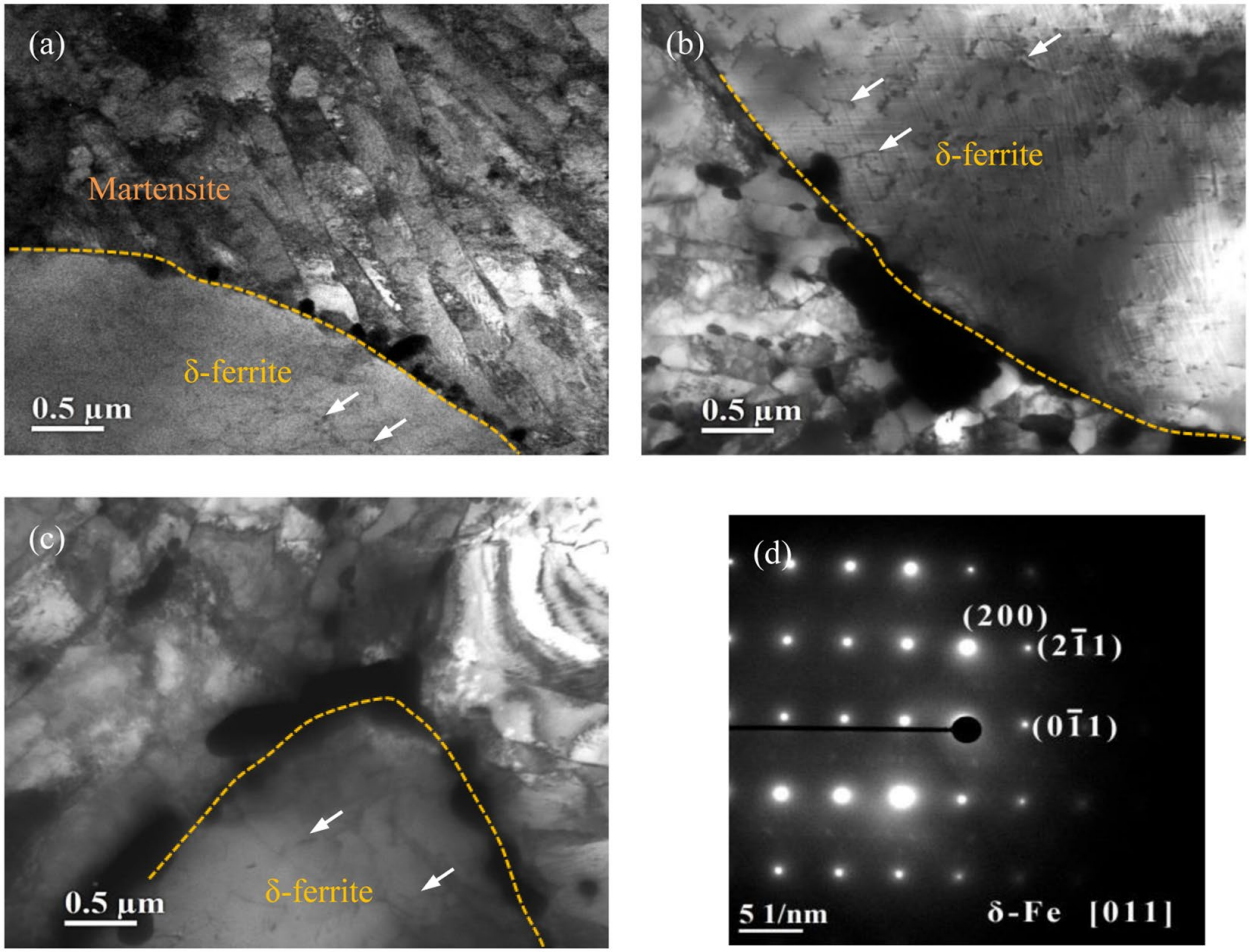
TEM images of precipitates located at the interface of delta ferrite and martensite in 9Cr-1.7W-0.4Mo-Co ferritic steel after isothermal aging for 100h (**a**), 500h (**b**), 1000h (**c**), and SAD result of delta ferrite (**d**).

Figure 2 shows TEM images of delta ferrite containing in 9Cr-1.7W-0.4Mo-Co ferritic steel after isothermal aging for different time. There are three obvious characters can be distinguished as follows:

(i) Some isolated dislocations (as marked by the light arrows in Fig. 2) were detected in the delta ferrite grains during isothermal aging, and the dislocation density kept to be increased with the increase in aging time. The atoms of dissolved alloying elements migrate from the interior to the boundary of delta ferrite, and form the precipitates located at the grain boundaries with the duration of isothermal aging, which means that many lattice vacancies are remained in delta ferrite grains.
(ii) The density of the precipitates located on the interface of martensite and delta ferrite was obviously higher, comparing to which located at the boundaries of martensite laths. This also can be attributed to that there is a relatively high density of dissolved alloying elements in delta ferrite, which is beneficial to the formation of precipitates, and also indicating that the particle size of precipitates at the interface should be larger.
(iii) Most of the particles of precipitates grew into martensite laths with the duration of isothermal aging, only partially small particles would inversely intrude into delta ferrite grains.

### Compositions of the precipitates

**Figure 3:**
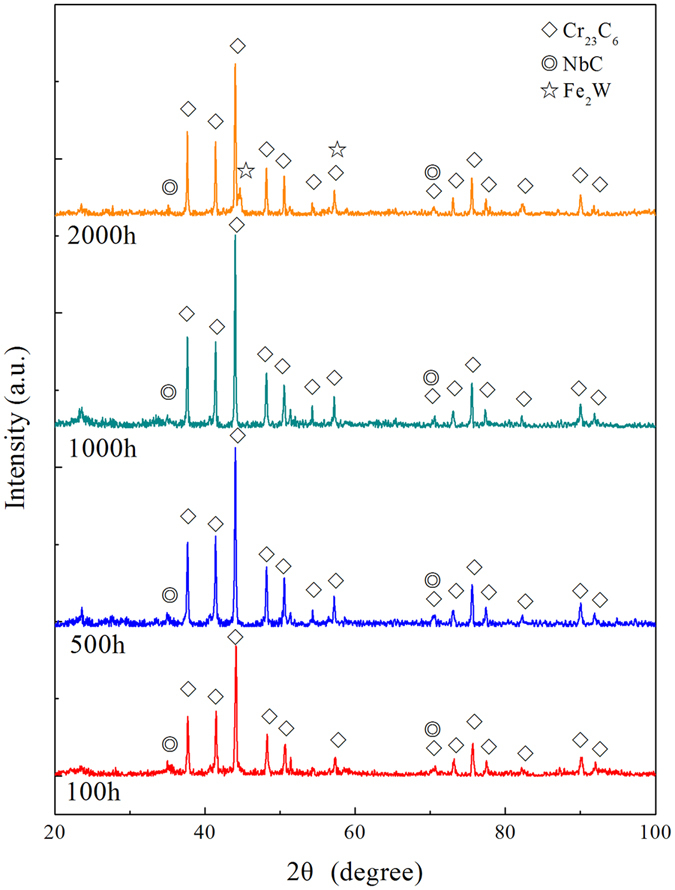
X-ray diffraction patterns of the precipitates obtained by electrolytic phase extraction in 9Cr-1.7W-0.4Mo-Co ferritic steel subjected to isothermal aging.

The powder of the precipitates in the aged 9Cr-1.7W-0.4Mo-Co ferritic steel obtained by EPE technology was analyzed using routine X-ray diffraction, as shown in Fig. 3. In the all XRD patterns, the remarkable peaks were clearly visible, and which could be identified as the Cr-rich carbide of an M_23_C_6_ type, *i.e*., Cr_23_C_6_ phase. Additionally, the peaks around 2-theta angles 35° and 71° represented an MX type precipitate enriched in Nb (NbC phase), which has also been detected from the XRD patterns. Fe_2_W Laves phase was not identified until the time of isothermal aging was extended to about 2000 h. In our previous research, the increase in W element content in some areas, such as grain boundaries and around M_23_C_6_ particles, is beneficial to match the formation of Fe_2_W Laves phase with the duration of isothermal aging^35^. Therefore, the migration and enrichment of W element on the grain boundaries or around the M_23_C_6_ particles during aging result in the formation of Fe_2_W phase.

**Figure 4:**
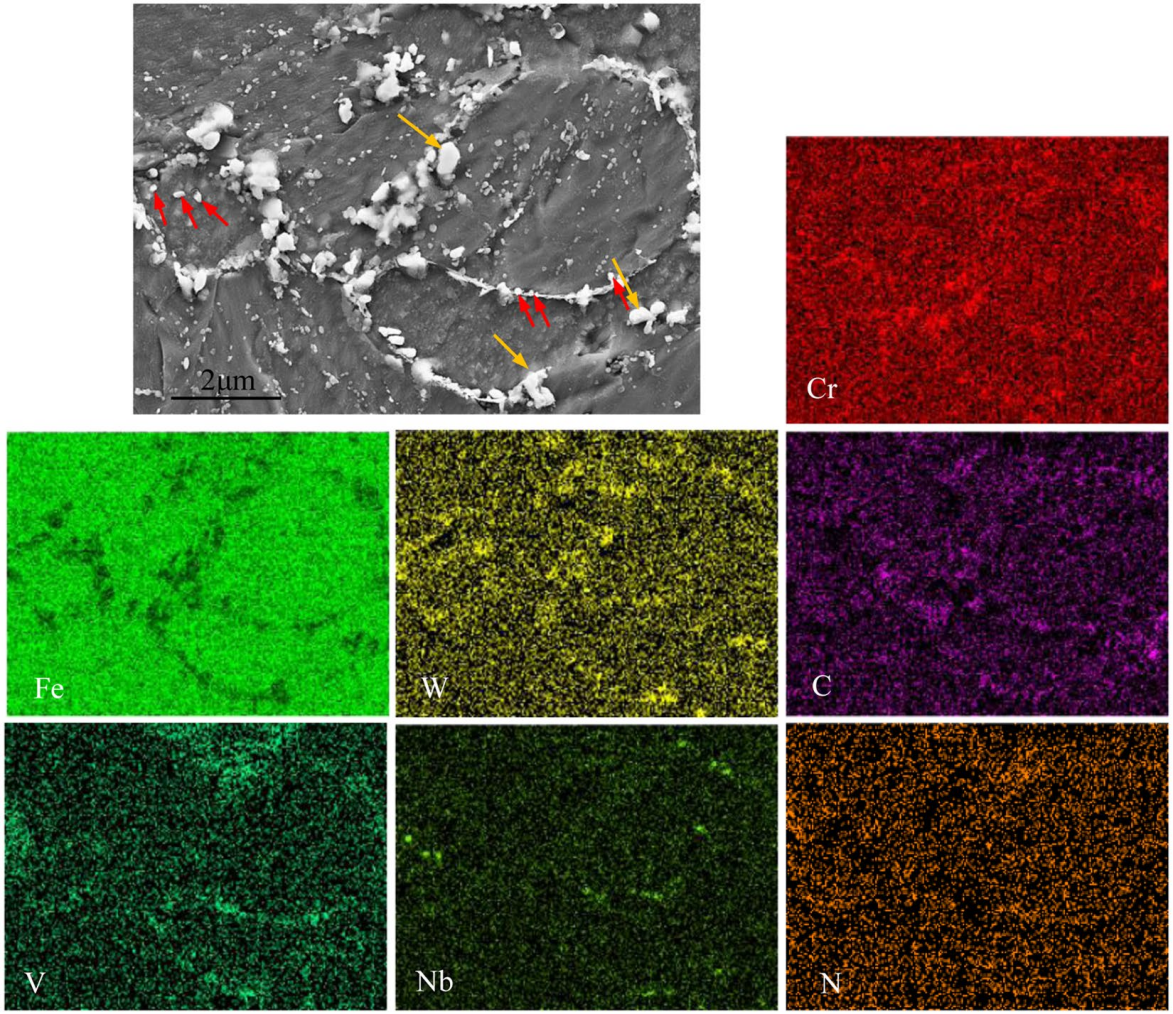
SEM and EDS analyses of alloying elements distributions in the microstructure of 9Cr-1.7W-0.4Mo-Co ferritic steel subjected to isothermal aging for 2000h.

The energy disperse spectroscopy (EDS) analyses of alloying elements distributions in the microstructure of the material subjected to isothermal aging for 2000 h are displayed in Fig. 4. Some subgrains can be observed with the duration of aging. The large and small precipitates particles were located on the subgrain boundaries and in the interior of subgrains. It can be seen that the alloying elements distributions among different particles are obviously heterogeneous. The high densities of Cr, C and W alloying elements distribute in the large particles with ellipsoidal and spherical shapes located at the subgrain boundaries, which indicates that the large particles can be identified as M_23_C_6_ precipitates enriched in Cr element. Several ellipsoidal particles enriched in W element and depleted in Cr element should be considered as Fe_2_W Laves phase (as shown by yellow arrows in Fig. 4). The content of Fe element in the Fe_2_W Laves phase was lower comparing to the Fe-matrix phase (martensite), which also should be noted.

**Figure 5:**
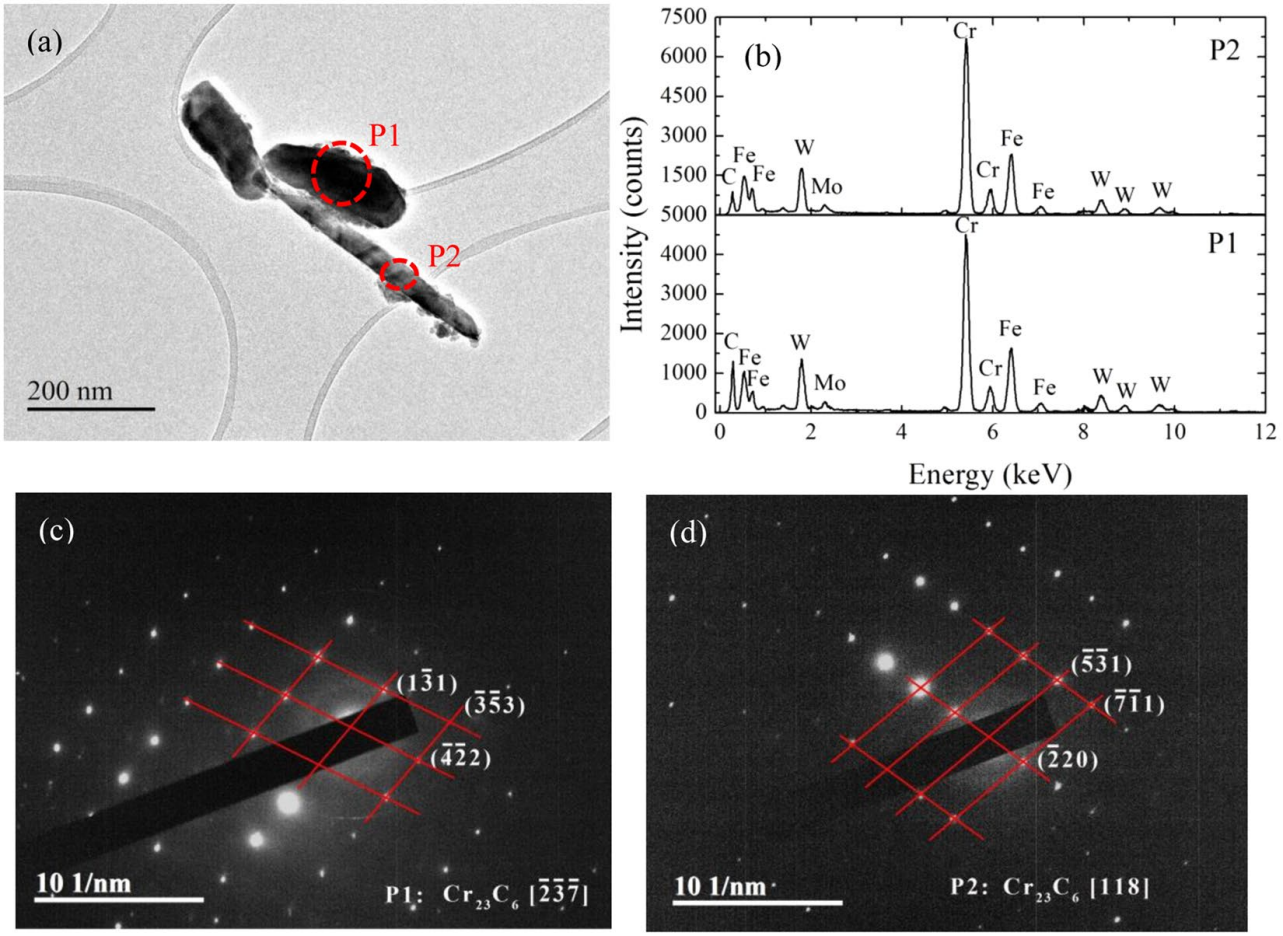
TEM image (**a**), EDS results (**b**) and the selected area diffractions marked with “P1” (**c**) and “P2” (**d**) of the precipitates in 9Cr-1.7W-0.4Mo-Co ferritic steel after isothermal aging for 100 h, showing M_23_C_6_ precipitates.

Figure 5a and b represent TEM micrograph and EDS results of the precipitates powder extracted from 9Cr-1.7W-0.4Mo-Co ferritic steel after 100 h aged. The ellipsoidally shaped particles with 200 nm and 65 nm in long axis and short axis, respectively, and a plate-shaped particle with 400 nm in length, can be obviously distinguished. The chemical compositions of these particles are similar to each other, containing high Cr and Fe contents with traces of W and Mo as shown in Fig. 5b. The selected area diffraction (SAD) patterns of the particles marked in Fig. 5a are displayed in Fig. 5c and d, identifying that both the particles with the two shapes are face-centered cubic structure (FCC), Cr_23_C_6_ precipitates. For the case of 9Cr–1Mo ferritic steel, it has already been established that the composition, size, and distribution of M_23_C_6_ precipitates are mainly dependent on ageing time and temperature: a longer time and higher ageing temperature are considered to induce equilibrium and hence a higher volume fraction of the precipitates^39^. Whereas, a long duration such as after 1000 h may mark the onset of carbide particle coarsening and the appearance of new brittle phases such as Laves and Z-phase^11, 20^.

**Figure 6:**
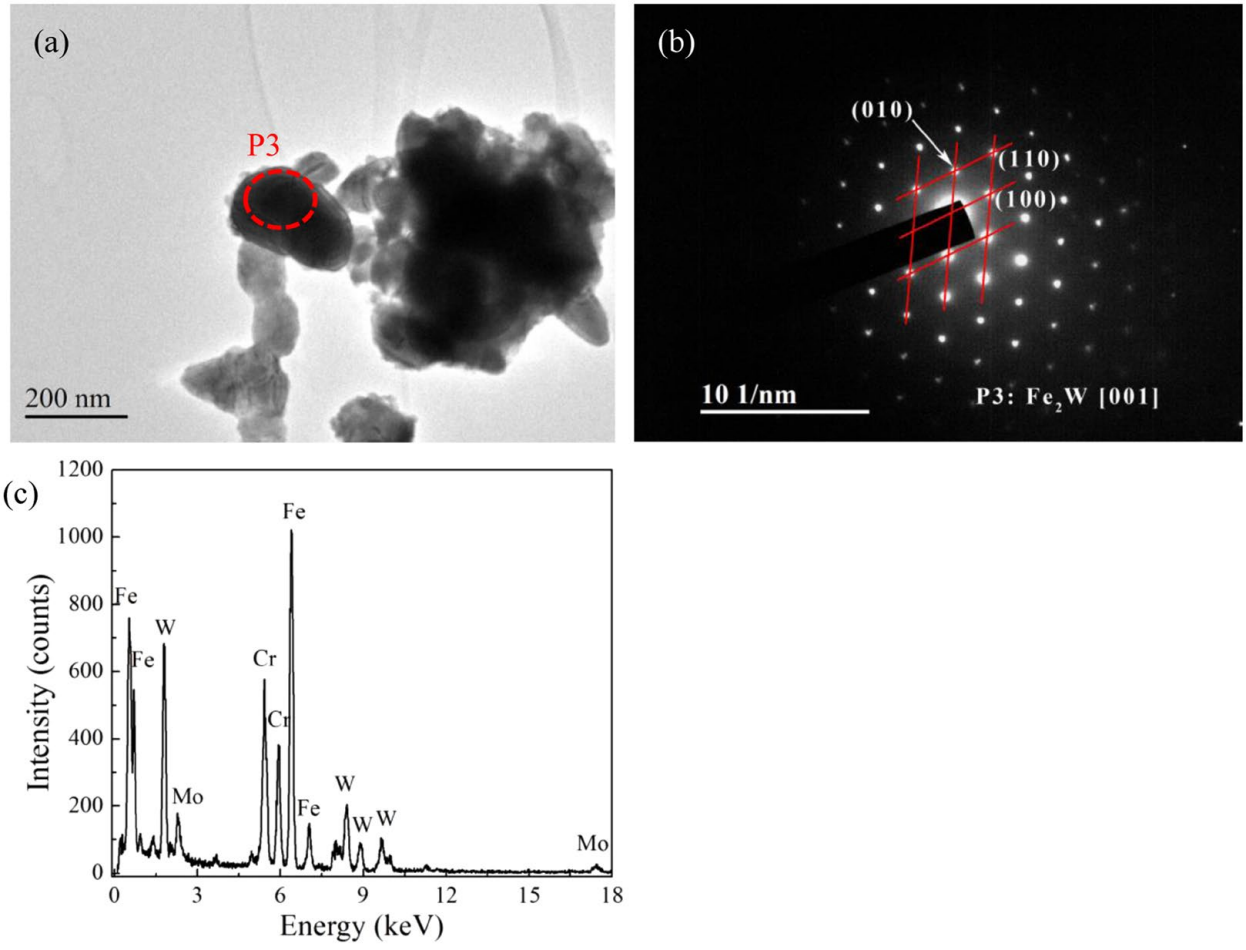
TEM image (**a**), SAD (**b**) and EDS result (**c**) of Laves phase (marked with “P3”) in 9Cr-1.7W-0.4Mo-Co ferritic steel after isothermal aging for 2000 h.

Figure 6 shows TEM analysis of Laves phase formed in the 9Cr-1.7W-0.4Mo-Co ferritic steel after isothermal aging for 2000 h. It should be pointed out that there is no Laves phase being found in other samples, such as aging for 1000 h, which means that the microstructure of the ferritic steel is still very stable during long-termed aging. The particle size of laves phase is measured to be ~ 200 nm, which is consistent with the literatures^40, 41^. Fe_2_W Laves phase can be identified using SAD and EDS methods. The EDS result indicates that Fe and W are dominant in the Laves phase with traces of Mo and Cr. One mechanism is that the nucleation of Laves phase precipitates at the Cr-depleted regions with relatively high W and Mo contents adjacent to M_23_C_6_ particles. The M_23_C_6_ particles are identified as a composition of (Fe, Cr, Mo, W)_23_C_6_ as we discussed above. Therefore, the alloying elements distributions in M_23_C_6_ particles with the durations of isothermal aging are analyzed by EDS, which can reflect the rearrangement of alloying elements and indicate the formed location of Laves phase, and the results are displayed in Table 1. The content of Cr increases from 53.87 wt.% to 63.31 wt.% in M_23_C_6_ precipitates with the increase in aging time from 100 h to 2000 h, W content inversely decreases from 19.25 wt.% to 11.86 wt.%, both of them show a rearrangement of alloying elements during isothermal aging. It means that the dissolved W in M_23_C_6_ phase will precipitate, and the precipitated W elements in the vicinity of M_23_C_6_ phase are beneficial to the formation of Fe_2_W Laves phase, and thus Laves phase is preferential to precipitate at the locations adjacent to M_23_C_6_ particles. In other words, for M_23_C_6_ phase during aging, the chemical compositions will be changed due to the formation of Laves phase, except the coarsening of the particle size during aging as reported in the literatures^42, 43^.

**Table 1:** Alloying elements contents in M_23_C_6_ precipitates of 9Cr-1.7W-0.4Mo-Co ferritic steel after isothermal aging (mass%).

	Fe	Cr	W	Mo	Nb	V
100h	21.48	53.87	19.25	4.32	0.22	0.86
500h	20.34	59.28	15.18	3.86	0.25	1.08
1000h	22.98	60.01	13.52	2.70		0.79
2000h	19.11	63.31	11.86	4.44	0.10	1.18

### Particle size of M_23_C_6_

Quantification of the particle size distributions of M_23_C_6_ phase was carried out based on TEM images of the EPE samples, and thus it is difficult to distinguish the location of M_23_C_6_ phase. To obtain sufficiently accurate statistics for the particle size distributions, the number of particles counted using computer-aided software was above 300. Employing Banerjee’ s method^44^, it is assumed that *A* represents the particle area, and the equivalent area diameter (EQAD), *d*_*A*_, can be given as:

1 $${d}_{A}=\sqrt{\frac{{4}A}{\pi }}$$

**Figure 7:**
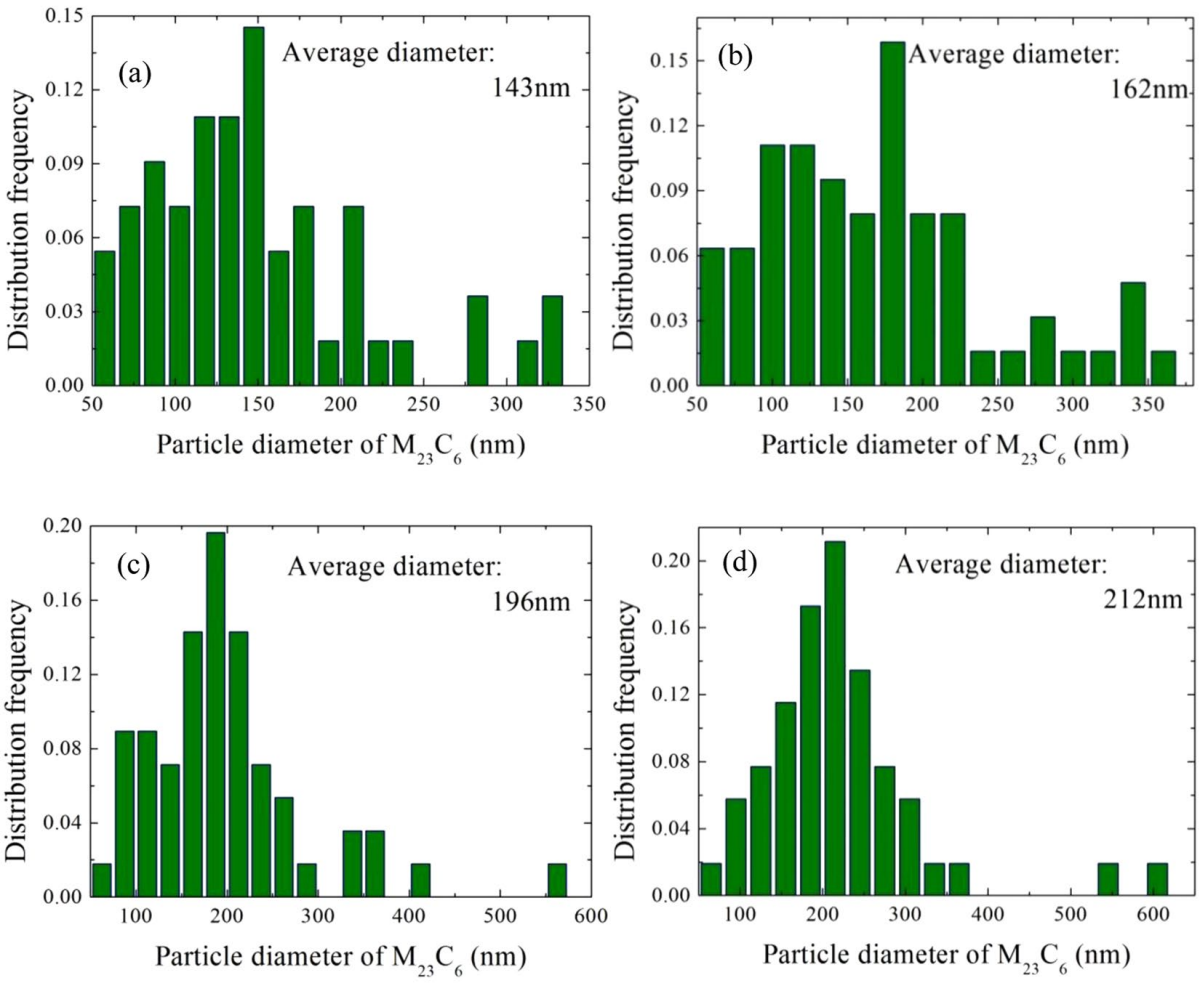
Distribution frequency as a function of particle diameter of M_23_C_6_ precipitates in 9Cr-1.7W-0.4Mo-Co ferritic steel after isothermal aging for 100h (**a**), (**b**) 500h (**b**), 1000h (**c**), 2000h (**d**), counting from TEM images of the EPE samples.

The particle diameter was obtained by using the measured particle area in Eq. (1). Hence, the obtained particle size distributions as a function of particle diameter are displayed in Fig. 7.

The changes in particle sizes of M_23_C_6_ precipitates can be considered to obey the Gaussian distribution in total. However, the distributions of particle sizes with ranges of 50~180 nm and 50~220 nm for the samples subjected to aging for 100 h and 500 h, respectively, are more uniform, comparing to the samples with a longer duration of ageing time (1000 h and 2000 h). With the increase in aging time to 1000 h, most of particle sizes increased to a range of 100~240 nm, indicating that M_23_C_6_ precipitates with small size are preferential to coarsen firstly, and the large M_23_C_6_ are relatively stable during short aging. The reason can be attributed to the distributions of alloying elements adjacent to M_23_C_6_ particles. The alloying elements, such as Cr and C around small M_23_C_6_ particles, can be considered to be homogeneous, which means that there are enough elements to migrate into M_23_C_6_ particles and promote the coarsening of precipitates. For large M_23_C_6_ particles, whereas, the existence of Cr depleted area will partly decrease the coarsening progress of M_23_C_6_ phase.

## Discussion

Theoretically, the alloying elements, formed the precipitates located on the interface of martensite and delta ferrite, are from the interior of delta ferrite, and thus the precipitate particles should be inclined to grow into delta ferrite. In fact, the original interface of martensite and delta ferrite has migrated into the delta ferrite with the diffusion of atoms, and advances ahead of the precipitates particles, which will leave the precipitates as isolated particles in the martensite. The migration of delta ferrite interface depends not only on the chemical composition, but also to some extent on the temperature which affect the diffusion rate of atoms as reported in the literatures^45–47^. When the aging temperature is to be above 873 K, it is considered that the migration of δ/α‘ interface is obviously ahead of the growth of the precipitates^45^. Hence, the precipitates were retained in martensite due to the migration of δ/α‘ interface during aging at 973 K which can be seen in Fig. 2.

In our previous works, the alloying elements comparisons between different phases have been accomplished, and the results showed that the contents of Fe in martensite, delta ferrite and precipitates were about 85.9 wt.%, 89.6 wt.% and 36.5 wt.%, respectively^35^. The highest content of Fe in matrix phase is easy to be understood. Besides, the MX-type precipitates enriched in Nb located on the subgrain boundaries and in the interior of subgrains also can be recognized, respectively (as shown by red arrows in Fig. 4). During aging/creep, Laves phase precipitated on grain or subgrain (martensite lath) boundaries has been proved that it will reduce the efficiency of grain boundary as a sink of dislocations, and suppresses the recovery of dislocations at grain boundaries^41, 48^. Nevertheless, the small particle size of Laves phase improves the pinning force of the migrations of dislocations and grain boundaries, and increases the stability of microstructure, which means that the role of Laves phase with this situation is similar to M_23_C_6_ and MX^49, 50^. As seen in Fig. 4, the average particle size of Laves phase is about 200~500 nm, which is same to M_23_C_6_ particles, indicating that the microstructure of the materials aged for 2000 h should be still stable due to precipitation strengthening of the precipitates.

M_23_C_6_ particle coarsening as a function of aging time has been studied in ferritic heat-resistant steels and other alloys^42, 51–53^. The particle coarsening rate for M_23_C_6_ phase so far follows the equations based on the classical Lifshitz-Slyozov-Wagner (LSW) theory which assumes supersaturated solid solution, misfit-free spherical particles, and one-rate controlling solute^51, 54, 55^. According to LSW theory, the coarsening kinetics of the particles for volume diffusion-controlled growth processes follows the law:

2 $${d}^{3}-{d}_{0}^{3}=kt$$

where *d* and *d*_*0*_ correspond to the average particle diameter at aging time *t* and the initial average particle diameter, respectively, and *k* is the coarsening rate constant with a formula as^56^:

3 $$k=\frac{8{C}_{e}{V}_{m}D\Omega }{9RT}$$

with *C*_*e*_ as the equilibrium concentration of atom in the matrix, *V*_*m*_ as the molar volume of the precipitates, *D* as the diffusion coefficient of atom in the matrix, *Ω* as the interfacial energy of precipitates/matrix, *R* as the gas constant, and T as the aging temperature in K.

**Figure 8:**
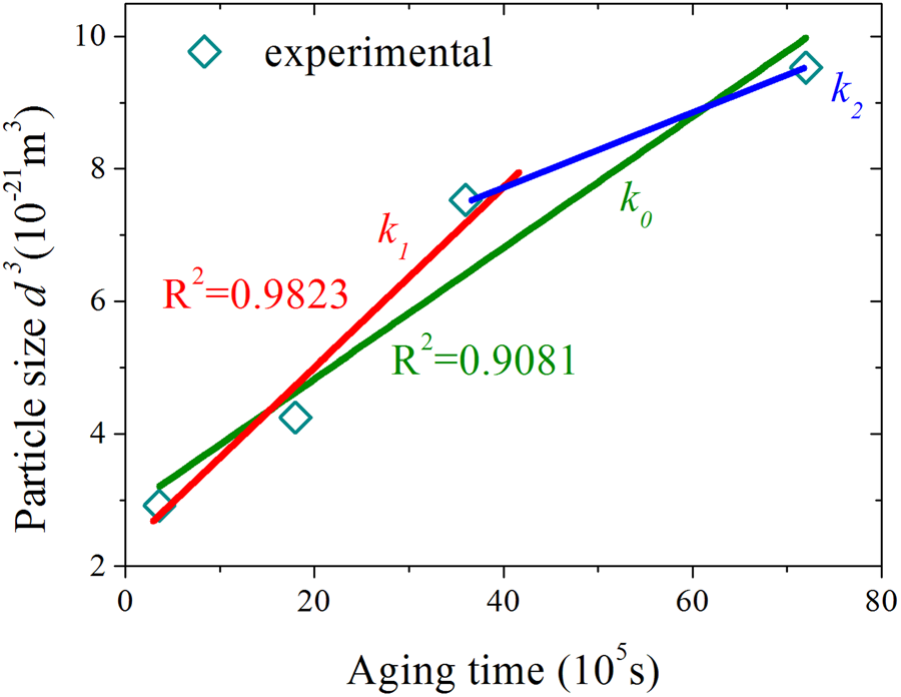
Coarsening of M_23_C_6_ particles in 9Cr-1.7W-0.4Mo-Co ferritic steel aging at 973 K, the cubic of measured average particle diameter and fitted curve as a function of aging time.

The average particle diameters of M_23_C_6_ phase aged at 100 h, 500 h, 1000 h and 2000 h are 143 nm, 162 nm, 196 nm and 212 nm as obtained from Fig. 7, respectively. Hence, the cubic of average particle diameter as a function of aging time in second (s) is shown in Fig. 8. The Laves phase is consisted with the same alloying elements, such as Fe, W, Mo and Cr, as M_23_C_6_ phase except for C. According to Gustafson^57^, the M_23_C_6_ coarsening should be higher if the Laves phase is not allowed to form, comparing to the situation that when the amount of Laves phase has an equilibrium amount. The other researchers inversely showed that the formation of Laves phase does not affect much the coarsening of M_23_C_6_^58^. There is no Laves phase can be found until increasing aging time to 2000 h in our work, it means that the influence of Laves phase on the M_23_C_6_ coarsening can be ignored under shorter aging time. A linear relationship between the cubic of average particle diameter and the aging time was determined for aging at 973 K, which approximately satisfies the predictions of LSW theory. The thus coarsening rate constant of M_23_C_6_ phase during aging was determined as *k*_*0*_ = 9.75 × 10^−28^ m^3^ s^−1^ from the slope (green fitted line). Whereas, the R-square value is bout 0.9081, indicating that the fitting result is not accurate. In fact, Laves phase is found in the sample aging for 2000 h, the initial nucleation/formation stage of Laves phase is still unknown. In addressing, two stages of M_23_C_6_ coarsening without and with the effect of Laves Phase are considered. Therefore, the first stage is under the assumption that the Laves phase was not allowed to form at all, *i.e*., from the aging beginning to about 1000 h, and the coarsening rate of M_23_C_6_ is about *k*_*1*_ = 1.21 × 10^−27^ m^3^ s^−1^ with a higher R-square of 0.9823. The second stage in our work should be considered to be from 1000 h to 2000 h with the coarsening rate of *k*_*2*_ = 4.52 × 10^−28^ m^3^ s^−1^. The results indicate that the formation of Laves phase will affect the coarsening of M_23_C_6_ phase.

The coarsening rates of M_23_C_6_ particle at the range from 873 K to 1023 K deduced from data in references and this work are listed in Table 2. As we know, the coarsening rate of M_23_C_6_ particle is mainly dependent on aging/creep temperature, and theoretically increases with the increase in temperature for the same materials, which is obviously identified in Table 2. The lowest coarsening rate is only 0.088 × 10^−29^ m^3^ s^−1^ in modified 9%Cr Steel with 1% Co at 873 K. Considering the existence of Laves phase at exposing temperature 973 K, the M_23_C_6_ coarsening is relatively low in 9Cr-1.7W-0.4Mo-Co ferritic steel, comparing to 12%Cr steel and H13 steel, indicating that M_23_C_6_ phase in our materials is stable at 973 K.

**Table 2:** Coarsening rates of M_23_C_6_ particle at the range from 873 K to 1023K obtained in references and this work.

Temperature (K)	Coarsening rate (×10^–29^m^3^s^−1^)	Materials
873	0.088	9%Cr Steel with 1% Co^36^
	0.12	P92 steel^5^
	1.03	P92 steel^57^
923	0.546	12%Cr steel^51^
	1.37	P92 steel^5^
	1.8	9% Cr Steel with 1% Co^36^
	25.3	P91 steel^5^
973	76.9	12%Cr steel contains Laves^13^
	*k*_*0*_ = 97.5	this work
	*k*_*1*_ = 121	
	*k*_*2*_ = 45.2	
	530	H13Nb steel^42^
	760	H13 steel^42^
1023	21.1	12%Cr steel^51^
	450	9% Cr Steel with 1% Co^36^

## Methods

Material used in the present work is a new modified ferritic heat-resistant steel, 9Cr-1.7W-0.4Mo-Co steel subject to being hot-rolled to 12 mm thick at 1373 K, and then tempered at 1023 K for 2 h. The chemical composition of the steel was determined by Inductive Coupled Plasma-Optical Emission Spectrometry (ICP–OES), and the thus obtained results are listed in Table 3. Further, the tempered steel was cut to several small pieces which were isothermally aged at 973 K holding for 100 h, 500 h, 1000 h and 2000 h, respectively. The aging tests were conducted in a vacuum tube furnace with a temperature tolerance of ± 5 °C, and a vacuum degree of −0.09 MPa in the furnace was controlled to avoid oxidation.

**Table 3:** Chemical compositions of the modified high Cr ferritic heat-resistant steel (mass%).

Element	C	Si	Mn	Cr	Mo	W	V	Nb	N	Co	B	Ti	Al
wt.%	0.05	0.21	0.44	9.81	0.43	1.73	0.22	0.07	0.03	1.21	0.0045	<0.01	0.014

Characterization of microstructure was realized by SEM (ZEISS, SUPRA-55) and TEM (JEOL, JEM-2010) at an accelerating voltage of 15 kV and 200 kV, respectively. The specimens for SEM were prepared by mechanical polishing, and then etching in a solution composed by iron trichloride (5 g), hydrochloric acid (20 mL) and distilled water (100 mL). Thin foils with 130~150 micrometers for TEM were prepared by a twin-jet electropolisher using a solution of 10% perchloric acid and 90% ethanol.

Electrolytic phase extraction (EPE) technology was employed to analyze the composition of the precipitates. Using EPE, the matrix phase was etched and dissolved in the solution, and only the precipitate particles were extracted. EPE of the samples at a current density of 120 mA/cm^2^ operated in a solution of 25% hydrochloric acid and 75% alcohol, and then the precipitates powder extracted from the samples were collected on the bottom of the flask after washing 3~5 times with alcohol. The compositions of the powder were identified by XRD analysis using Cu Kα radiation at room temperature, with a step size of 0.02° and observed by TEM.

## Conclusions

The precipitates in the 9Cr-1.7W-0.4Mo-Co ferritic heat-resistant steel subjected to isothermal aging at 973 K were investigated using SEM, TEM and XRD. The M_23_C_6_ particle coarsening as a function of aging time is also described. The conclusions are summarized as follows:

1. The isolated dislocations were detected in delta ferrite interior, and increase with the increase in aging time. Both the density and the particle size of the precipitates on δ/α‘ boundaries are obviously larger than other locations, and the migration of δ/α‘ boundaries leads to the retained precipitates in martensite.
2. Cr_23_C_6_, NbC and Fe_2_W precipitates are recognized based on XRD, and Fe_2_W-Laves phase can only be found as duration of aging time to 2000 h. The precipitated W elements in the vicinity of M_23_C_6_ phase are beneficial to the formation of Fe_2_W Laves phase, which shows that Laves phase is preferential to form at the locations adjacent to M_23_C_6_ particles.
3. M_23_C_6_ precipitates with small particle size are preferential to coarsen firstly, and the large M_23_C_6_ are relatively stable during short aging.
4. The total coarsening rate of M_23_C_6_ precipitates is 9.75 × 10^−28^ m^3^ s^−1^, but it changes to 1.21 × 10^−27^ m^3^ s^−1^ without the formation of Laves phase, and 4.52 × 10^−28^ m^3^ s^−1^ with consideration of Laves phase, respectively, indicating that the coarsening of M_23_C_6_ precipitates depends on the formation of Laves phase.
